# Intuitive Feelings of Warmth and Confidence in Insight and Noninsight Problem Solving of Magic Tricks

**DOI:** 10.3389/fpsyg.2016.01314

**Published:** 2016-08-31

**Authors:** Mikael R. Hedne, Elisabeth Norman, Janet Metcalfe

**Affiliations:** ^1^Department of Psychosocial Science, Faculty of Psychology, University of BergenBergen, Norway; ^2^Department of Psychology, Columbia UniversityNew York, NY, USA

**Keywords:** intuition, insight, magic, aha! experience, problem solving, metacognitive feelings, warmth ratings, confidence ratings

## Abstract

The focus of the current study is on intuitive feelings of insight during problem solving and the extent to which such feelings are predictive of successful problem solving. We report the results from an experiment (*N* = 51) that applied a procedure where the to-be-solved problems were 32 short (15 s) video recordings of magic tricks. The procedure included metacognitive ratings similar to the “warmth ratings” previously used by Metcalfe and colleagues, as well as confidence ratings. At regular intervals during problem solving, participants indicated the perceived closeness to the correct solution. Participants also indicated directly whether each problem was solved by insight or not. Problems that people claimed were solved by insight were characterized by higher accuracy and higher confidence than noninsight solutions. There was no difference between the two types of solution in warmth ratings, however. Confidence ratings were more strongly associated with solution accuracy for noninsight than insight trials. Moreover, for insight trials the participants were more likely to repeat their incorrect solutions on a subsequent recognition test. The results have implications for understanding people's metacognitive awareness of the cognitive processes involved in problem solving. They also have general implications for our understanding of how intuition and insight are related.

## Introduction

Experiences of insight may occur in many different domains—both in cognitive activities like perception, language comprehension, and problem solving, as well as during moments of self-awareness in clinical psychological settings (Kounios and Beeman, 2014). The focus of the current paper is on insight experiences in a special kind of problem solving during which the individual is trying to figure out how a magic trick was done. Sometimes, as in other kinds of problem solving, such solutions are characterized by their sudden appearance, and by a special feeling state, often referred to as an Aha! experience (e.g., Topolinski and Reber, 2010; Salvi et al., 2016). In line with focus of the research topic, we ask whether problem solving of magic tricks that occurs with or without the Aha! experience is differentially reflected on intuitive, metacognitive feelings during and after the solution attempt. This would in turn shed light on whether the two types of problem solving differ in the availability of relevant conscious knowledge.

The question of how intuition and insight are related follows from existing debates concerning the involvement of automatic/unconscious vs. controlled/conscious processes in insight problem solving. To illustrate this debate, take two models that both focus on the processes that lead up to the change in problem representation preceding insight. According to *progress monitoring theory*/*satisfaction progress theory* (MacGregor et al., 2001) problem solving involves the conscious, step-by-step monitoring of one's problem solving behavior. Two mechanisms are proposed for how this monitoring occurs. One is mental simulation, which involves that the problem solver tries to look ahead and predict the consequences of future moves. The other is evaluation of prospective moves against an internal criterion, which makes it possible to estimate the likelihood of success or failure. For both mechanisms, the emphasis is on conscious and intentional planning, monitoring, and evaluation. In contrast, according to *representational change theory* (Ohlsson, 1992; Knöblich et al., 1999), insight problem solving initially involves the construction of an erroneous problem space. Representational change can then occur through constraint relaxation, i.e., the release of unnecessarily constraining assumptions, or chunk decomposition, i.e., deconstruction of perceptual chunks into smaller features, which may in turn be recombined into more productive representations. According to this model, neither the erroneous problem representation nor the mechanisms that resolve it, need to involve intentional, conscious deliberation. Instead, they are assumed to be characterized by automatic and unconscious processes. Other theories that focus on unconscious mechanisms in problem solving include those of Smith and Kounios (1996), and Topolinski and Reber (2010). The latter theory focuses on the interplay between conscious and unconscious mechanisms in problem solving, and provides a framework for understanding how the phenomenology of insight can be understood as the conscious correlate of processing fluency caused by a sudden appearance of the solution. It should be added that one could also assume a continuum of understanding, from shallow to deep, in which intermediate levels of understanding are possible. It could also be that the extent to which a problem representation may be understood in this way would depend on the complexity of the problem.

Among researchers who acknowledge the role of unconscious processes in insight problem solving, there is disagreement over whether insight occurs through a sudden/discontinuous or gradual/continuous process. Theories that focus on the mechanisms involved in cognitive restructuring (e.g., Kounios and Beeman, 2014) would often imply that insight is a product of non-deliberate, unconscious processing that is independent of conscious, analytic thought (Smith and Kounios, 1996). An alternative is to regard insight as resulting from a gradual, more *continuous* process. The idea is that, over the course of the problem solving attempt, the problem representation changes from being unconscious/vague to becoming conscious/verbalisable. Importantly, this latter view does not imply any sudden, qualitative shift in information-processing (e.g., Bowers et al., 1990; Zander et al., 2015). Central to either view is that the subjective experience of insight would involve the activation of relevant unconscious/implicit knowledge. For example, Bowers et al. (1990) referred to an insight/hunch as involving a behavioral preference for a certain solution before this solution can be verbalized/justified. Similarly, Kounios and Beeman (2014) argued for the involvement of unconscious knowledge in insight problem solving by referring to findings demonstrating that subliminal priming may facilitate insight problem solving. A different hypothesis that seems compatible with a discontinuous view is the one by Topolinski and Reber (2010), who argued that the subjective experience of insight reflects increased perceptual fluency associated with the sudden activation of a solution. Thus, even though it is commonly agreed that insight would involve implicit/unconscious knowledge, there is disagreement about the processes by which such knowledge gives rise to the subjective experience of insight.

Furthermore, when people solve incrementally by satisficing, or getting to a “good enough” answer, the answer itself may be less stable than when they solve by insight. Novick and Sherman (2003) refer to insight solutions as “pop-out” solutions. By the Gestalt view of problem solving (see Kounios and Beeman, 2014), insight solutions have a crystallized quality, resulting from a restructuring of an unstable organization into a new *stable* structure. The stability of the solution, and both the correctness of this new structure and the individual's confidence in it and willingness to change it, will be of interest in the present research.

One way to get a better understanding of the relationship between intuition and insight is to measure intuitive, metacognitive feelings associated with insight vs. noninsight solutions to a set of problems, and to measure the relationship between such feelings and aspects of problem solving. Whereas the relationship between subjective feelings and unconscious knowledge has been extensively studied in relation to other forms of implicit cognition, including implicit learning (e.g., Dienes and Scott, 2005; Norman and Price, 2015), the question of how subjective feelings relate to objective performance at the different stages of insightful vs. noninsightful problem solving is still under-explored. A demonstration of whether and how unconscious knowledge is related to insight requires a clearer understanding of how subjective feelings relate to objective performance in problem-solving situations. The focus of the current paper is on how the two forms of problem solving differ in terms of the relation between intuitive feelings and objective performance during and after problem solving, which would provide an important contribution to the ongoing debate on the cognitive mechanisms underlying insightful problem solving.

Metcalfe and Wiebe (1987) studied the relation between prospective intuitive feelings and objective performance by asking participants to provide warmth ratings at regular intervals whilst the person was working on each problem. The question was whether warmth ratings would predict problem solving differently depending on whether the problems were multistep problems/puzzles (e.g., the Tower of Hanoi task), or vignette descriptions previously demonstrated to give rise to insight solutions (e.g., the “water lilies problem”). Metcalfe and Wiebe found that warmth ratings increased gradually before people produced the correct solutions to the first type of problem (referred to as “incremental” problems), but did not increase much before people gave the correct solutions to the latter kinds of problems (referred to as “insight” problems). The authors argued that the difference in phenomenology accompanying insight and incremental problem solving could be used to define insight.

However, a limitation of this and other classical paradigms for studying insight vs. noninsight problem solving relates to the fact that they make use of two different sets of tasks. When, in studies like that of Metcalfe and Wiebe (1987), participants are presented with 2 sets of different problems that are predefined to be associated with either insight or not, behavioral or self-reported differences between the two could also be attributed to factors other than those related to information-processing differences associated with the presence or absence of insight. For example, tasks could differ in terms of difficulty, motivation/engagement, the number of steps needed for solution, or involvement of prior knowledge (see also Bowden, 1997; Bowden et al., 2005; Kounios and Beeman, 2014, for similar arguments). In addition, it has recently been argued that the use of pre-defined insight problems may be problematic because correct solutions to these problems are not always characterized by Aha! experiences (Danek et al., 2016).

Danek et al. (2013, 2014a,b) developed a novel experimental paradigm to counter these limitations. Rather than presenting participants with different sets of problems that were pre-defined to be associated with insight or not, their experimental stimuli were a series video recordings of magic tricks. Their assumption was that magic tricks can potentially be solved with or without insight. They argued that magic tricks can sometimes be solved with sudden insight that occurs as a result of constraint relaxation. However, they may also be solved in a step-by-step manner, which involves that the person systematically considers different possibilities (Danek et al., 2014a). The researchers therefore asked participants to report, for each suggested solution, whether or not the solution was associated with the experience of insight. As predicted, Danek et al. found that some solutions were associated with insight whereas others were not. Importantly, they also found that the two types of solution were associated with measurable differences on a number of dependent variables. Insight solutions were more likely to be accurate, occurred after fewer presentations, and were associated with higher levels of confidence than noninsight solutions. Furthermore, in a different paper reporting results from the same experiment (Danek et al., 2013), it was found that insight solutions were also remembered more accurately. Danek et al. interpreted these results as supporting the idea that problem solving characterized by insight is qualitatively different from problem solving without insight. It should be noted that since such a procedure does not make claims about which problems are more likely to be solved with or without insight based on, e.g., assumptions about the necessary problem solving steps involved. Instead, the focus is on the subjective experience of insight/Aha! In the remainder of the paper, we refer to problem solving characterized by this form of subjective experience as “insight problem solving.”

Importantly, such a procedure makes it possible to explore the relationship between intuitive feelings (of, e.g., warmth and confidence) and objective indices of problem solving across the two types of solution, without the possible confounding influence of task differences. Thus, the procedure can be used to address whether the two forms of problem solving differ in terms of conscious availability of relevant knowledge. However, the specific procedure used by Danek et al. also had some limitations. First, their definition of insight specifically stated that it is characterized by high confidence. Participants were told that an Aha! experience would be characterized by feeling “relatively confident that your solution is correct” (p. 662). To circumvent the potential risk of demand characteristics, in the experiment that we present here, we took care to *not* include any information concerning confidence in the definition we gave participants about what comprised an insight solution. Moreover, in the earlier work of Danek et al., the measure of solution time could be criticized for low precision. Because their measure was the number of presentations (from 1 to 3) rather than absolute solution time in seconds, the true difference in solution time within a single category might be larger than between categories. We standardized the duration of each video, and used milliseconds as the measurement of solution time^1^. Furthermore, they did not systematically assess the relationship between confidence and accuracy, which could have given insights into the conscious status of activated knowledge. To explore this we measured the confidence related to the accuracy for each solution type. Additionally, their sole measure of intuitive feelings was retrospective confidence, and they also did not include any measurement of intuitive feelings *during* the solution attempt. In the present study we evaluate intuitive feelings of nearness to the solution before the solution is given, in a manner similar to Metcalfe and Wiebe's warmth ratings. Finally, they had no measure of the stability of the solutions once they had been given. If insight solutions were more crystallized than noninsight solutions it would be expected that people would be unlikely to change them. Therefore, the tendency to hold on to the suggested solution was measured by including a multiple choice task giving several options for possible solutions.

### Aims of current study

The main aim of the current study was to explore whether the relationship between intuitive feelings and behavioral measures differed for solutions characterized by insight vs. solutions that were not, when the to-be-solved problems were magic tricks. This would in turn contribute to our understanding of the availability of conscious knowledge in the two forms of problem solving. We both asked participants to provide prospective warmth ratings while working on each problem, as well as confidence ratings after having provided a suggested solution. Based on previous findings (Danek et al., 2014b), we predicted that the two types of solution would differ with respect to solution time, accuracy, and confidence. If our subjective measures of confidence and warmth were found to be more strongly related to objective indices of problem solving for noninsight than insight problems, this would support the view that insight to a larger extent involves implicit/unconscious knowledge. Although our study alone is not designed to directly test whether insightful problem solving reflects a continuous or discontinuous process, a similar pattern of equally predictive warmth and confidence ratings across the two types of solution would be compatible with a continuous view of insight. We were also interested in whether the insight solutions were more stable than the noninsight solutions, and this was tested by comparing the stability between the suggested solution and the subsequent multiple choice. In conjunction with the multiple choice task participants would also report their decision strategy, where one of the options described having chosen the alternative most closely resembling the already suggested solution.

## Methods

### Participants

Fifty-one students (14 male, 37 female), aged 19–31 (*M* = 21.81, *SD* = 2.55) were recruited from the University of Bergen (The Faculties of Humanities, Law, Mathematics and Natural Sciences, Medicine and Dentistry, and Psychology). Each participant received a gift card of NOK 150 (about 18 USD) as a compensation for participating. The total duration of the experiment was between 50 and 70 min, depending on how much time participants spent on individual trials. The research was conducted in accordance with the stipulations of the declaration of Helsinki, and conformed to the regulations of the Norwegian Data Protection Official for Research.

### Materials

The task was programmed in E-prime 2.0 (Schneider et al., 2002a,b) and displayed by a 19″ monitor. All instructions were in Norwegian, and all written instructions relating to the experimental procedure were presented on screen. Participants were tested in groups of 3–5 in individual cubicles in a psychology testing room. The post-experimental questionnaire and instructions were presented in paper format.

We reviewed the list of magic tricks presented in Danek et al. (2014b), and selected tricks based on a number of criteria. These included timing of individual tricks and variability across tricks in terms of effect and method. A magic trick consists of an initial situation, a magic moment, and a revelation (de Ascanio, 1964/2005), and for a trick to be selected it had to be structured so that it was possible to clearly present all these three phases within the time frame of 15 s. The different tricks selected should also cover a variety of different basic magic effects, e.g., production, vanish, transformation, penetration (Fitzkee, 1944/1989). Additionally, the methods used to accomplish the different effects should vary across tricks. Some of the magic tricks used similar methods to accomplish different magical effects, whereas other magic tricks used different methods to accomplish similar effects. Most of the magic tricks were accomplished using methods specific to those magical effects, making sure the problems to be solved were all different. All methods used should be possible to describe in a simple and straightforward fashion using relatively few words. Each magic trick was presented as a problem solving task with little or no use of misdirection or superfluous gestures. Of the 32 magic effects selected, 20 were used in the study conducted by Danek et al. (2014b).

**Figure 1:**
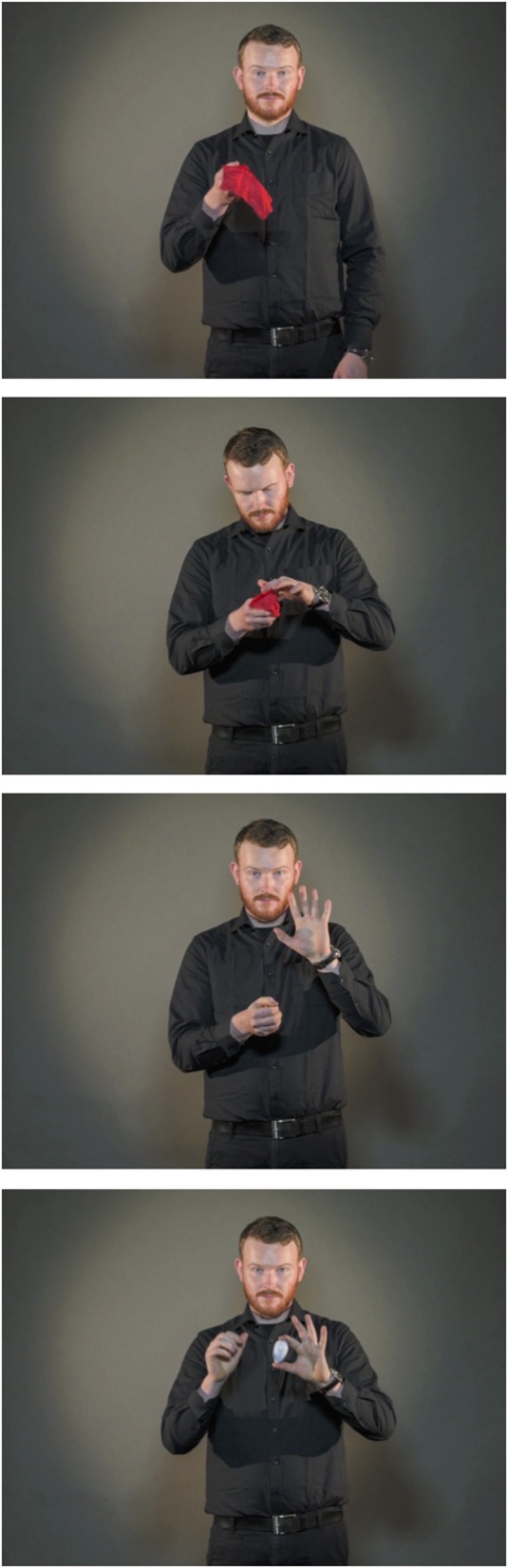
**Picture sequence illustrating the magic trick Silk to egg (Example 1)**. The full clip is available at https://www.youtube.com/watch?v=_jE25LbLaoQ.

**Figure 2:**
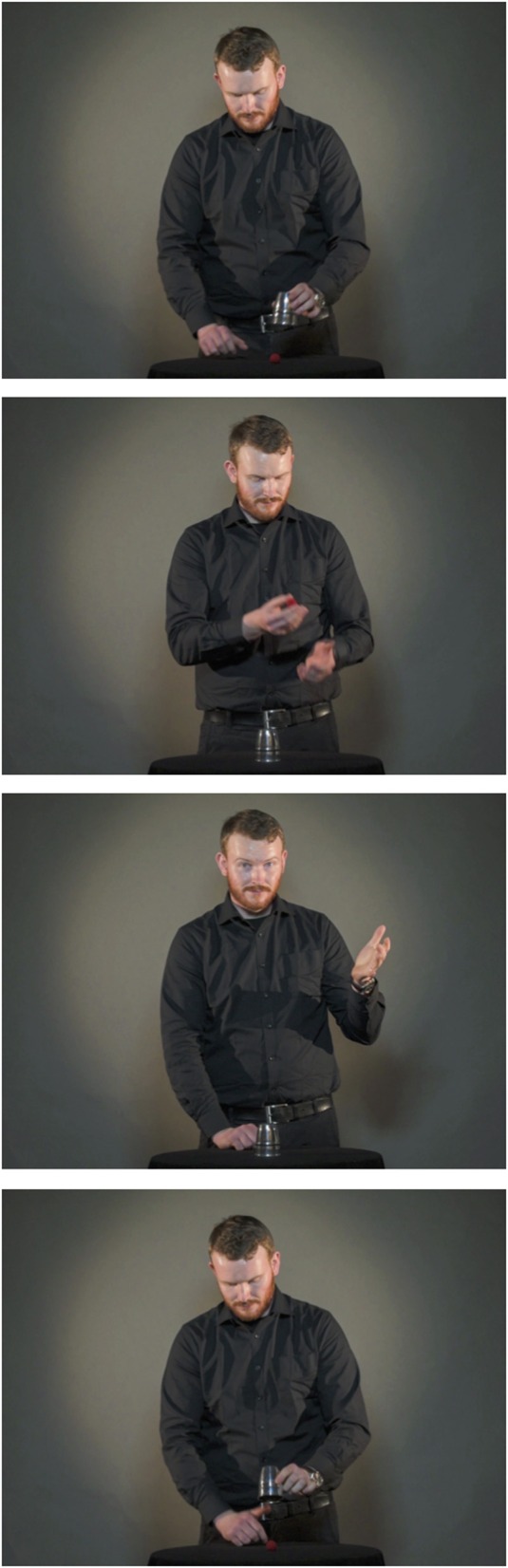
**Picture sequence illustrating the magic trick Chop cup (Example 2)**. The full clip is available at https://www.youtube.com/watch?v=YTvTFNnwDEg.

**Figure 3:**
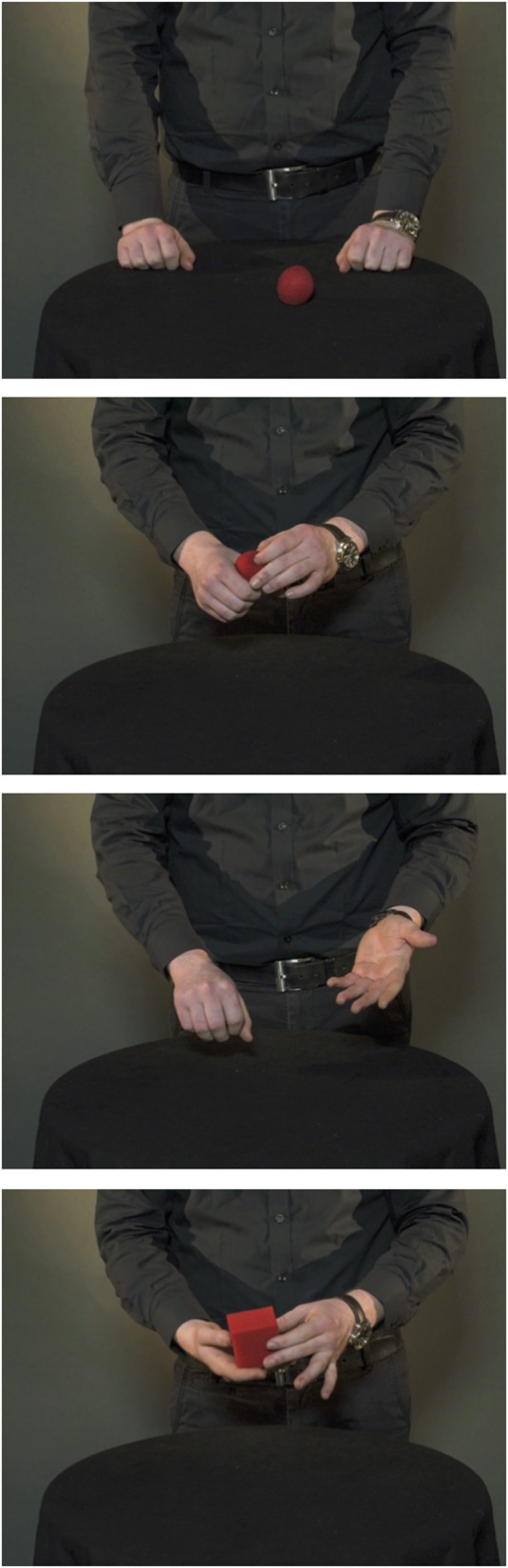
**Picture sequence illustrating the magic trick Ball to cube (Example 3)**. The full clip is available at https://www.youtube.com/watch?v=VqNYrADykUk.

On each of the 32 trials, a video was presented that displayed a professional magician performing a magic trick. The videos were filmed in a photographic studio and each video clip had a duration of 15 s. The full clips of three of the tricks are available online, and are also illustrated by picture sequences in Figures 1–3 (Example 1: https://www.youtube.com/watch?v=_jE25LbLaoQ/ Figure 1; Example 2: https://www.youtube.com/watch?v=YTvTFNnwDEg/ Figure 2; Example 3: https://www.youtube.com/watch?v=VqNYrADykUk/ Figure 3). As different magic tricks require different points of focus from the spectator, 13 of the videos were filmed viewing the magician standing upright (See Example 1), 6 displayed the magician standing behind the table (See Example 2), and 13 displayed the magician's hands and a tabletop (See Example 3). A full list describing all the 32 magic tricks is provided in the Appendix.

### Procedure

#### Instructions

At the start of the experiment, participants were given verbal instructions relating to the overall procedure as well as to our definition of an Aha! experience. This was described as a solution appearing “out of nowhere” and as being different from other / previously suggested solutions. Furthermore, it was instructed that if they could explain the entire reasoning process leading up to the solution, this would not be considered an Aha! experience. The definition was similar to the one used by Danek et al. (2013, 2014a,b), with the only difference being that we did not include reference to confidence. Before proceeding to the experimental procedure, each individual participant was asked by the experimenter whether they had understood the definition and whether they had any further questions.

#### Practice trials

Participants were first given a practice trial where they were shown a short and unrelated video clip before being asked to click on a visual analog scale (VAS). On the second practice trial they were to watch the unrelated video clip once more and were instructed to abort the video at a certain point by pressing the spacebar. Finally they were shown what would be the duration of the warmth rating (WR) scale in the following procedure (4000 ms), to inform them of how much time they would have to answer the warmth rating.

#### Problem solving task

The videos of the 32 magic tricks were presented in a different randomized order for each participant. Each trial consisted of the initial presentation of the magic trick followed by a WR display where the participant was to indicate perceived closeness to the solution. WR was reported using mouse click on a VAS consisting of a bar colored with a blue (“cold”) to red (“warm”) gradient. The WR scale would disappear after 4000 ms if no response was given. The first WR scale was followed by a break of 11,000 ms before another WR scale was shown and then followed by another presentation of the video. Each video could be displayed a maximum of 3 times. Every trial sequence would thus include a maximum of 3 presentations of the given video clip, 2 breaks, and a total of 5 WRs between each of these presentations/breaks.

**Figure 4:**
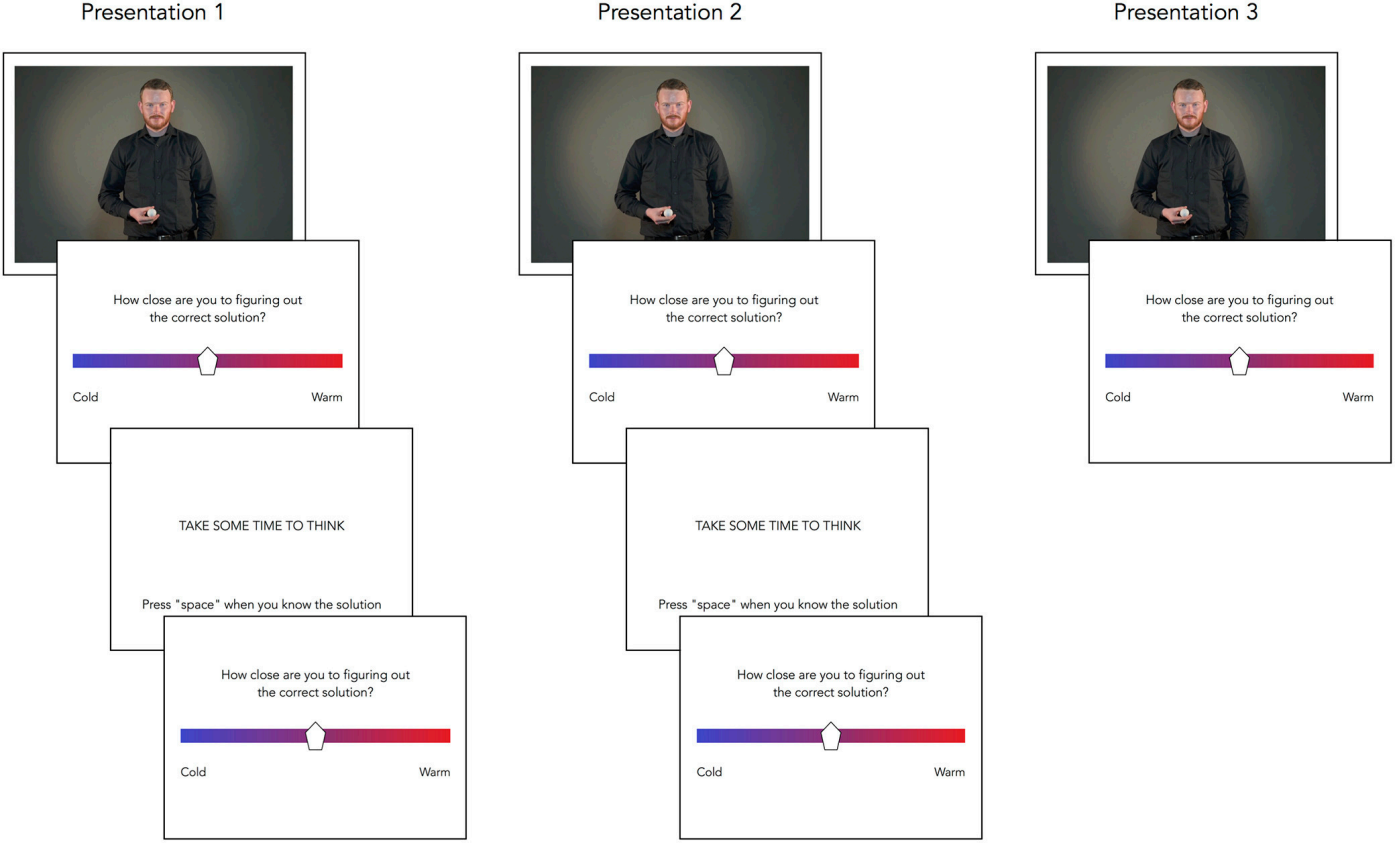
**A picture sequence of a trial of the main problem solving task**. Each trial consisted of up to 3 presentations of the video, up to 5 warmth ratings, up to 2 breaks.

Participants were instructed to press the spacebar once they knew the solution for the magic trick being displayed. Pressing the spacebar would abort the ongoing sequence, and this could be done at any point after the first video presentation had been completed. If the participants did not press the spacebar, the sequence would run out for the aforementioned maximum duration. This procedure is depicted in Figure 4.

In all cases, both when the participant would abort the sequence or if it ended by timeout, the participant was presented with the question “Did you have an Aha! experience?” They answered this by indicating “yes” or “no”. An on-screen text box then appeared, in which they were to type in the solution for the magic trick, or write “don't know” if they did not have any hypotheses for how the trick was done. After having written the solution they were to report their confidence related to the suggested solution. This was done using a VAS similar to the WR scale with a bar colored in gradients from light gray (“not at all confident”) to dark gray (“totally confident”).

#### Recognition

After reporting the confidence related to the written solution, participants were given a multiple choice task of four possible solutions of which one was the correct solution. This was followed by a confidence rating similar to that used in the problem solving task, but was now related to the chosen alternative. They were finally asked to report the strategy used for arriving at the chosen alternative, with the alternatives being: “After looking at and comparing all the four alternatives I chose the one I thought to be the most probable,” “The moment I saw one of the alternatives I knew it had to be the correct one,” “The alternative I chose was the one most similar to my written solution,” “I felt equally uncertain of all the alternatives and chose one at random.” The procedure for the problem solving task and recognition was then repeated until all 32 videos had been viewed.

Participants did not receive any feedback about the accuracy of their chosen solution for neither the written description nor the recognition-task.

#### Questionnaires

After completing the 32 trials the participants were first given a questionnaire asking if they knew anyone who had, or had themselves, been doing magic as a hobby or professionally at any point in their lives. They were also asked if they had knowledge about magic beyond what they perceived to be the average.

## Results

### Rating the accuracy of solutions

Initial data analyses excluded single trials where the participants had reported that they did not know how the magic trick was done or where no response was given. Two raters (both professional magicians) scored all the remaining solutions independently on a 4-alternative scale (completely incorrect-1, mostly incorrect-2, mostly correct-3, completely correct-4), with the cutoff for correct/incorrect being 2/3. The 4-alternative scale was used only for the purpose of scoring, making it evident which items required the most thorough discussions. Inter-rater reliability measured using Cronbach's alpha was 0.911. As a rule of thumb, if a magic trick involved several minor effects (such as the vanish and reappearance of a ball), all of these had to be accounted for if the solution provided were to be rated as correct. Trials where the raters had scored differently were discussed case-wise if the ratings were different with regard to incorrect (1 or 2) vs. correct (3 or 4). For the remaining analyses accuracy of solutions were measured as dichotomous. Trials rated 1 or 2 were given the value 0, and trials rated 3 or 4 were given value 1.

Time was measured in milliseconds, and warmth and confidence were measured in whole values ranging from 1 to 100.

### Filtering of data

Several other cases were excluded for different reasons. Cases where the response given was more than one single solution were excluded from the analysis both for instances where one of the suggested solutions were correct and in cases where neither of the suggested solutions were correct. This was also valid for cases where the participant would not understand the magic effect. Cases where the participant did not abort the procedure (i.e., timeouts) were also excluded from the further analyses as these were considered errors of omission (Salvi et al., 2016). Data from 8 of the participants were excluded altogether as they did not report any Aha! experiences. Trials involving one of the magic tricks (“Three Card Monte”) were excluded across all participants as no one reported the correct solution. Finally, several single trials were excluded in cases where participants reported, either in the text box during the procedure or in the post-experimental questionnaire, that they had prior knowledge of how the magic trick was accomplished. The reason for this filtering was to make sure that the two groups of solution types did not differ in any way which might cause erroneous results (e.g., neither timeout trials nor trials with the response “don't know” would occur for insight trials). After excluding trials not fulfilling the set criteria (661), a total of 971 trials were left for the remaining analyses.

### Insight vs. noninsight solutions

Of the included trials (*N* = 971), 29% were reportedly solved using insight, whereas 71% of the trials were not. There was substantial variability in the frequency with which different tricks were solved with or without insight. To illustrate, example video 1 (https://www.youtube.com/watch?v=_jE25LbLaoQ, see also Figure 1) was the problem most frequently solved with insight. In contrast, example video 3 (https://www.youtube.com/watch?v=VqNYrADykUk, see also Figure 3) was the problem least frequently solved with insight.

We will now give an example of how a single magic trick could be solved both with and without insight. In the magic trick “Chop Cup” (https://www.youtube.com/watch?v=YTvTFNnwDEg/ Figure 2), a ball is taken from under a cup, vanished, and then reappears under the cup. For this particular magic trick, an understanding of the premise involves understanding that the ball to vanish is not the same as the one reappearing under the cup (i.e., the trick involves using two identical balls). This understanding may take the form of an Aha! experience. If one has understood this core premise, one can then deduct from this how the first ball is vanished and the second one is produced. A noninsight route to the same solution would be to first realize that the magician does not place the ball to be vanished in his hand before showing the hand empty. This, however, will not explain how the ball can reappear under the cup. Only by then understanding that the ball to appear under the cup is in fact different from the one vanishing will the spectator have understood the premise.

A series of *t*-tests were conducted with self-reported solution type (insight vs. noninsight) as the independent variable, and accuracy (correct/incorrect), solution time, and confidence in the written solution as the dependent variables, respectively. We expected insight solutions to be associated with higher accuracy, shorter solution time, and higher confidence. As predicted, there was a significant difference in solution accuracy in each task for solutions reported as insight (*n* = 281, *M* = 0.57, *SD* = 0.50) and solutions reported as noninsight (*n* = 690, *M* = 0.37, *SD* = 0.48); *t*_(506.9)_ = 5.78, *p* < 0.001, *d* = 0.51. The average time spent before aborting the procedure showed a non-significant trend in the predicted direction between trials characterized by insight (*M* = 38.23, *SD* = 18.69) vs. noninsight trials (*M* = 40.56, *SD* = 19.53); *t*_(969)_ = 1.705, *p* = 0.089, *d* = 0.10. This borderline trend becomes significant (*p* < 0.05) with a one-tailed *t*-test.

### Warmth ratings

Analyses comparing the development of warmth rating across time for insight vs. noninsight trials only included trials containing 3 or 4 points of measure. Trials where WR was reported on all 5 points were already excluded due to the omission criterion. It was assumed that participants may sometimes wait for a short time between figuring out the solution and aborting the procedure^2^. To avoid this possible confounding influence, the first WR rating was compared with the second last (rather than the last) rating. This corresponds to the procedure used by Metcalfe and Wiebe (1987), who compared the first WR rating to the last rating before the rating given with the answer. Trials containing less than 3 points of data were therefore also excluded from these particular analyses. Warmth ratings were analyzed in terms of two types of scores that corresponded to “differential” and “angular” warmth measures (Metcalfe and Wiebe, 1987). Differential warmth was calculated by subtracting the first value from the last, similar to Metcalfe and Wiebe's procedure. This raw score could range from -99 to 99. Angular warmth was calculated by dividing differential warmth by seconds. This is based on a similar reasoning as both methods will measure development in warmth controlled for time. We expected to find a higher value for differential and angular warmth rating on trials not associated with insight.

A set of *t*-tests showed no significant difference in differential warmth ratings between trials with solutions characterized by insight (*n* = 50, *M* = 2.92, *SD* = 23.26) and noninsight (*n* = 162, *M* = 0.30, *SD* = 19.52); *t*_(210)_ = 0.79, *p* = 0.429, *d* = 0.12. There was also no significant difference in angular warmth ratings between trials with solutions characterized by insight (*M* =.41, *SD* = 2.60) and noninsight (*M* = −0.004, *SD* = 0.29); *t*_(49.36)_ = 1.12, *p* = 0.266, *d* = 0.22. Although, as noted above, the last warmth rating probably should not be included in the analysis, when we did include it, the means for insight and noninsight solutions with the different analyses were 12.93 (*SD* = 18.87) and 11.65 (*SD* = 16.38) (differential warmth, *t*_(239.38)_ = 0.72, *p* = 0.47, *d* = 0.07), and 0.24 (*SD* = 0.36) and 0.22 (*SD* = 0.31) (angular warmth, *t*_(239.84)_ = 0.82, *p* = 0.42, *d* = 0.08), respectively. Thus, these findings contrast with the earlier findings of Metcalfe and Wiebe.

### Confidence

There was a significant difference in mean confidence between insight (*M* = 78.32, *SD* = 20.35) and noninsight (*M* = 68.95, *SD* = 23.96); *t*_(606.8)_ = 6.17, *p* < 0.001, *d* = 0.50. This finding is important as participants in previous studies were explicitly instructed that they would be more confident on insight than noninsight solutions. Our instruction did not mention confidence, and yet participants were, in fact, more confident about insight solutions.

In order to compare the relationship between confidence and accuracy separately for the different solution types, two sets of analyses were conducted, one of which used mean values (i.e., trial based) and the other signal detection statistics (i.e., participant based). First, *t*-tests were conducted examining each solution type respectively, with accuracy treated as if it were an independent variable. For insight solutions confidence was significantly higher for correct (*n* = 160, *M* = 81.34, *SD* = 17.16) than incorrect trials (*n* = 121, *M* = 74.32, *SD* = 23.41); *t*_(210.97)_ = 2.78, *p* < 0.01, *d* = 0.34. The same was true for noninsight solutions, where mean confidence was higher for correct (*n* = 254, *M* = 74.31, *SD* = 22.73) than incorrect trials (*n* = 436, *M* = 65.83, *SD* = 24.14); *t*_(688)_ = 4.55, *p* < 0.001, *d* = 0.36.

The relationship between confidence and accuracy in the two conditions was compared using the signal detection theory (SDT) statistic Az (Macmillan and Creelman, 2004; Norman and Price, 2015). This is calculated from performance across the different values of the rating scale, and corresponds to the area under the SDT ROC curve. This area expresses the “probability of being correct for a given level of confidence” and can be regarded as indicative of the individual's metacognitive ability (Song et al., 2011, p. 1789). An Az score of 1 indicates perfect discrimination between correct and incorrect answers, and an Az score of 0.5 indicates random responding. Note that Az scores need to be calculated for each individual subject; thus, the following analyses are subject-based rather than trial-based.

Comparing the Az scores between insight (*M* = 0.56, *SD* = 0.28) and noninsight trials (*M* = 0.63, *SD* = 0.18) in the 33 participants who had a valid Az score for both types of trials^3^, there was no significant difference between the two groups *t*_(32)_ = 1.06, *p* = 0.297, *d* = 0.30. There was also no significant difference from random responding (0.5) in mean Az score for trials associated with insight (*M* = 0.57, *SD* = 0.28)^4^; *t*_(33)_ = 1.51, *p* = 0.142, *d* = 0.25. For trials not associated with insight, though, mean Az scores were significantly higher than what would result from a random assumption, (*M* = 0.64, *SD* = 0.17); *t*_(41)_ = 5.43, *p* < 0.001, *d* = 0.82. Thus, when a person solved with insight they seemed unable to judge whether they were right or wrong, whereas they could make this distinction when they produced a noninsight response.

### Recognition

To evaluate whether people were differentially persevering with the responses they had produced when they had experienced insight or not, we separated trials on which participants indicated that they chose the alternative most similar to their written solution, from those on which they claimed to have recognized the chosen alternative using any other strategy. Reported decision strategy was recoded as a dichotomous variable (“The alternative I chose was the one most similar to my written solution”—1; “other strategies”—0). Comparing the two sets of strategies, there was a significant difference between trials associated with insight (*M* = 0.72, *SD* = 0.45) vs. noninsight attributions (*M* = 0.61, *SD* = 0.49); *t*_(969)_ = 3.37, *p* = 0.001, *d* = 0.23. When analysing trials where the written solution was correct, there was no significant difference between insight (*M* = 0.79, *SD* = 0.41) and noninsight (*M* = 0.78, *SD* = 0.41); *t*_(412)_ = 0.25, *p* = 0.80, *d* = 0.02. For trials where the written solution was incorrect, there was a significant difference between insight (*M* = 0.63, *SD* = 0.49) and noninsight (*M* = 0.51, *SD* = 0.50); *t*_(196.58)_ = 2.41, *p* = 0.017, *d* = 0.24, indicating that participants had a stronger tendency to hold on to incorrect solutions for trials recognized by insight than noninsight.

## Discussion

In the present study we explored whether the relationship between metacognitive, “intuitive” feelings and objective indices of problem solving differed for insight vs. noninsight solutions when the to-be-solved problems were magic tricks (cf. Danek et al., 2013). The aim was to increase our understanding of the conscious availability of relevant knowledge in the two forms of problem solving, thus contributing to ongoing debates regarding conscious vs. unconscious processes in problem solving. A methodological aim was to explore the applicability of magic tricks as a problem solving task.

### Accuracy and solution time

In line with previous findings, insight solutions were more likely to be correct than noninsight solutions. This result is consistent with Danek et al.'s findings (2014b) and with notion that insight nearly always predicts correctness (Ohlsson, 1992; Salvi et al., 2016). In the present study, several of the trials solved by insight were incorrect. A reason for this could be that the participants were ignorant to magic tricks and their methods, as well as to how the responses were scored. A response was considered correct only if it described the actual method used to accomplish the magic effect. It might be that if a provided solution is feasible (Danek et al., 2014b), albeit incorrect, the participant has still understood the basic *premise* of the problem, without being aware of the particular details of the method itself. That said, for most of the problems presented in the current study, only one solution was possible given the presented context.

Contradicting the results of Danek et al. (2014b), there was little evidence supporting the hypothesis that solution time would be shorter for insight trials compared to noninsight trials. This could be due to differences in experimental design and time measurements, as the present study featured videos all with a duration of 15 s, and milliseconds as measurement for solution time. In the experimental procedure developed by Danek et al. (2013), the videos lasted between 6 and 80 s, and solution time was measured as the number of presentations for each video (1–3). Considering that the magic moment and revelation in a magic trick usually takes very little time and happens at the end of the entire magic trick, the initial situation of the magic trick (de Ascanio, 1964/2005) could then be used to contemplate on how to solve the problem at hand. For shorter videos, participants would then in be given less time to solve the problem.

It could be argued that limiting each video clip to 15 s limits the design to feature simple magic tricks. However, even with this constraint, one of the magic tricks (Three Card Monte) was not solved by any of the participants. Using more complex magic tricks as problems could also give rise to what is perceived as several possible solutions (Tamariz, 1988), whereas the magic tricks used would most often only have one possible solution, and as such be comparable to a puzzle.

### Warmth ratings

Contrary to predictions, there were no differences in the development of warmth ratings for insight vs. noninsight solutions. One possible explanation is that the two types of solution were preceded by the same underlying problem-solving processes (Bowers et al., 1990; Zander et al., 2015). However, it could also be related to our measurement procedure. Due to the aforementioned exclusion criteria, several trials were dismissed when measuring warmth. Even though participants could report warmth up to 5 times for each trial, only trials including 3 or 4 warmth ratings were used in the analyses, resulting in the exclusion of 70% of all trials. 3 or 4 ratings constitute relatively few data points in this form of analysis, and by comparison, the original study by Metcalfe and Wiebe (1987) allowed for up to 40 warmth ratings per problem.

Another salient difference between Metcalfe and Wiebe's (1987) study and the present one is that in the former, participants had to be 100% confident in their answer before providing it. People were not free to give an answer with low confidence, as they were in the present study. As those authors noted and as is consistent with the present data, when a person is working on a problem they may come to a tentative solution without high confidence. In order to be allowed to provide that (wrong) answer in Metcalfe and Wiebe's experiment, they would have to convince themselves that the answer was correct, or maybe good enough, and increase their confidence rating about that answer. This increase in confidence due to allowing that a solution that is not a perfect solution is actually good enough—the acceptance of a ‘satisficing’ solution—might itself have accounted for the incrementality seen in their noninsight condition, and also seen when people were solving insight problems but produced the wrong answer. Indeed, high confidence on insight problems just before the answer actually predicted that a mistake would be produced (Metcalfe, 1986), as if people might have been going through a self deceptive process of convincing themselves that a wrong answer was acceptable. (Note, that in the present study they would have been able to simply give the wrong response with low confidence).

### Confidence ratings

Although insight and noninsight trials did not differ in terms of warmth ratings, they differed in terms of confidence ratings given *after* arriving at the solutions. This indicated that, cognitively, they were not identical. The results showed that confidence reflected solution accuracy more precisely for noninsight than insight trials. Confidence ratings have previously been used to measure awareness of knowledge used in problem solving (Metcalfe, 1986; Metcalfe and Wiebe, 1987) as well as in other types of cognitive tasks, including implicit learning (Shanks and St. John, 1994; Dienes and Berry, 1997).

In the present study, insight trials were characterized by an overall stronger conviction that one's solution was correct, as well as overall more accurate responding. This is in line with the claim by Topolinski and Reber (2010) that the experience of insight is accompanied by a feeling of being right. However, confidence was in fact *less* predictive of solution accuracy for insight when this relationship was compared for correct vs. incorrect trials within individual participants. The relatively stronger correspondence between confidence and accuracy on noninsight trials, combined with the fact that confidence did not predict accuracy above chance level for insight trials, could be interpreted as indicating that participants had more metacognitive awareness of the accuracy of the provided solution on trials not characterized by insight. The contention that there was a difference between the two types of problem solving is further supported by the self-reported decision strategies for recognition judgments. Participants perseverated more with their incorrect solutions for insight than noninsight trials, indicating they were more likely to adjust their solution for the latter.

The finding is also compatible with the idea of high-confidence responses reflecting higher-quality mental representations, and with Danek et al.'s (2013) findings that insight solutions were associated with better long-term recall. Even though there was no support for the hypothesis that access to metaknowledge preceding the solution was different for insight vs. noninsight, the results involving intuitive feelings and decision strategies occurring *after* arriving at the solution, indicated that the two types of problem solving did indeed reflect qualitatively different processes.

### Insight as reflecting unconscious knowledge

The aim of including metacognitive measures of warmth and confidence was to make it possible to draw inferences about the conscious availability of relevant knowledge in the two forms of problem solving (Norman and Price, 2015). Whereas, a correspondence between confidence and accuracy indicates that behavior is influenced by conscious knowledge, the lack of such correspondence is normally taken to indicate unconscious knowledge (Dienes and Berry, 1997).

Our finding that confidence was less predictive of accuracy on insight trials could therefore indicate that such trials were characterized by relatively less conscious awareness of relevant knowledge. For example, insight trials may involve less access to *conscious fragment knowledge* and/or *informative cues* related to the provided solution (e.g., noticing a detail in the scene that one may use as a basis for subsequent hypothesis testing). Alternatively, it could be that insight trials are associated with a deeper understanding of the *premise of the problem*, but that this understanding is not fully available to conscious introspection/verbalisation at the time confidence is rated. If this is true, one could assume that when having an Aha! experience, participants first understand the core premise of the magic trick, and then “fill in the blanks” (Metcalfe and Wiebe, 1987; Smith and Kounios, 1996). The higher accuracy for insight trials could thus indicate that participants in these cases are more likely to have understood the problem “more fully”, i.e., to have a more complete understanding of the problem^5^ (Dominowski and Dallob, 1995), whereas for noninsight solutions they may be more likely to have understood and solved one piece of the problem whereas other parts are left unsolved. The relatively lower confidence for (incorrect) noninsight solutions could then reflect that on noninsight trials, participants were metacognitively aware that their knowledge/understanding was partial as opposed to complete. In contrast, on insight trials participants may intuitively have felt that they had understood the problem more fully. However, if they lacked conscious access to the details of this knowledge, they would be less able to metacognitively monitor its correctness, resulting in a lower correspondence between confidence and accuracy.

In sum, the confidence results suggest that problem solving by insight at least partly reflects unconscious knowledge. In other words, insight reflects more than just conscious, step-by-step monitoring (MacGregor et al., 2001). Instead, the results seem more compatible with theories that emphasize automatic/unconscious cognitive processes in insight problem solving (e.g., Ohlsson, 1992; Smith and Kounios, 1996; Knöblich et al., 1999; Topolinski and Reber, 2010).

Even though this conclusion would be stronger if also supported by the results involving warmth ratings, there are several reasons why the warmth measurement in the current experiment was not sensitive to possible differences in the cognitive processes preceding insight vs. noninsight solutions. Future studies should measure warmth in ways that avoid these limitations, which are accounted for in more detail earlier.

### Insight as resulting from a continuous or discontinuous process

Insight has been viewed as either a product of a discontinuous (e.g., Kounios and Beeman, 2014) or continuous process (e.g., Bowers et al., 1990; Zander et al., 2015), and a better understanding of whether insight is preceded by intuitive feelings or whether it reflects a sudden shift in information-processing is clearly needed. The fact that insight solutions were associated with higher accuracy and confidence compared to noninsight solutions, and also displayed a weak trend for shorter solution time, could be taken to support a discontinuous view. The same holds for the findings that insight solutions were characterized by a weaker correspondence between confidence and accuracy, and a stronger tendency to hold on to the provided solution, than noninsight solutions. Even though these findings are related to what happens *after* the insight has occurred, they could nevertheless be used to argue for qualitative differences between the two types of problem solving. In contrast, the lack of difference in warmth ratings between insight and noninsight trials does lend support to the continuous view. Thus, together the results do not give a clear answer to the question of continuity. In order to provide a clearer answer to this question, future studies should include additional measures of intuitive feelings and a larger number of measurement points. More specifically, additional points of data for intuitive feelings that occur before arriving at the solution would increase the experiment's sensitivity in reflecting possible differences in the development of warmth ratings across the two types of trials.

### Limitations and future directions

Even though self-reported Aha! experience is by many regarded as indicative of insight problem solving (e.g., Bowden et al., 2005; Bowden and Jung-Beeman, 2007; Sandkühler and Bhattacharya, 2008; Danek et al., 2016), there is still a concern that what we here classify as insight solutions were not necessarily arrived upon exclusively through insight, or that noninsight solutions did not purely reflect an incremental process. Instead, some solutions may have been reached through a combination of both. The fact that the problems to be solved were all from the same set of tasks may even have increased the possibility that participants used largely similar strategies appraising each problem across different trials. This could be due to the aforementioned issue relating to participants receiving feedback, as well as a consideration that magic tricks as a problem solving task cannot necessarily be separated into categories of purely insight or incremental problems. If this was the case, this may to a certain extent explain why warmth ratings were not more different across the two types of trials. However, the fact that the two types of solution were subjectively experienced by participants as being different, and the fact that participants tended to hold on to their suggested solutions more strongly on high-confidence insight trials, both go against this possible criticism.

## Author note

We would like to express our gratitude to Mats Svalebjørg for performing the magic tricks and contributing to the scoring of the solutions. We would also like to thank the people at Myreze for their contribution in filming the magic tricks.

## Author contributions

This study was conducted within a student scholarship project granted to MH. The supervisor for this project was EN. MH and EN contributed to the research design, data analysis, interpretation, and critical revision of the manuscript. MH programmed the experiment, and had the main responsibility for data collection and handling, as well as drafting the manuscript. JM contributed to the data analysis, interpretation, and revision of the manuscript.

## Funding

The project was supported by a student research grant from the Faculty of Psychology at the University of Bergen and a grant from Skibsreder Jacob R. Olsen og hustru Johanne Georgine Olsens legat (grant no. 2016/11/FOL/KH).

### Conflict of interest statement

The authors declare that the research was conducted in the absence of any commercial or financial relationships that could be construed as a potential conflict of interest.

## Footnotes

^1^In our view, the advantages of controlling for duration are larger than the possible limitations associated with this procedure (e.g., that the complexity of tricks cannot be varied within a single experiment).

^2^This assumption was confirmed through a questionnaire distributed to a subset of participants after completion of the experiment.

^3^For Az to be calculated, there needs to be at least 1 response in each category (correct vs. incorrect).

^4^The means and SD's differ from the above analyses due to casewise exclusion in the paired sampled t-tests.

^5^for a description of how this can manifest, see the description of the magic trick “Chop Cup” in the section “Insight vs. noninsight solutions” under Results.

## Appendix

**Table:**

**Trick name**	**Magic effect**
Appearing cane	A silk handkerchief transforms into a cane
Appearing pole	A long pole is pulled out of a suitcase
Appearing silk^*^	A silk handkerchief appears out of thin air
Ball to cube^*^	A ball turns into a cube
Chop cup	A ball disappears from the hands and reappears under a cup previously shown empty
Coin through silk^*^	A coin penetrates a silk handkerchief
Color changing cards 1	A queen of clubs transforms into a queen of spades
Color changing cards 2^*^	Two playing cards, one in a glass and the other under a handkerchief, switch places
Color changing knives^*^	A yellow knife changes color to red
Floating cigarette	A cigarette floats under the magician's control
Floating match	A matchstick floats over a playing card
Fork and spoon^*^	A spoon and a fork switch places
Ghost card^*^	A playing card is seen turning over by no visible aid
Linking rings	Two metallic rings are linked and unlinked
Match through match^*^	Two matchsticks are seen penetrating each other without breaking
Matrix	Four coins move from separate corners of a table to a single corner
Moving coin	Two coins are shown, one of which travels from one hand to the other
Multiplying balls^*^	One white ball turns into two and then back to one
Orange to apple^*^	An orange transforms into an apple
Paper to money^*^	Blank sheets of paper are transformed into banknotes
Pen through banknote^*^	A pen is pushed through a banknote without the banknote taking any damage.
Rubik's cube^*^	An unsolved Rubik's cube is solved after being tossed into the air
Shuffled/unshuffled	The cards are seen mixed face-up/face-down, before all facing the same way
Silk to egg^*^	A silk handkerchief transforms into an egg
Stick from purse	A long stick is pulled out of a small purse
Three card monte^*^^†^	Three playing cards are seen, two of the cards switch places
Torn and restored playing card^*^	A playing card is torn and then restored
Vanishing bottle^*^	A beer bottle is placed in a paper bag and vanishes
Vanishing card case	A deck of cards in a case is seen placed into a black container, and then vanished
Vanishing coin^*^	A coin vanishes from the magicians hand and then reappears
Vanishing glass^*^	A drinking glass is covered and vanished
Water to ice^*^	Water is poured into a cup and then turned to ice cubes

* This or a similar magic trick was also reported being used by Danek et al. (2014b). The presentation of the trick as well as the method used to achieve the desired effect might be different.

† This magic trick was excluded from the analyses because no participant provided a correct response.
